# Epidemiological risk factors for clinical malaria infection in the highlands of Western Kenya

**DOI:** 10.1186/s12936-019-2845-4

**Published:** 2019-06-24

**Authors:** Walters M. Essendi, Anne M. Vardo-Zalik, Eugenia Lo, Maxwell G. Machani, Guofa Zhou, Andrew K. Githeko, Guiyun Yan, Yaw A. Afrane

**Affiliations:** 1 Biological Sciences Department, Egerton University, Egerton, Kenya; 2 The Pennsylvania State University, 1031 Edgecomb Avenue, York, PA 1740 USA; 3 Department of Biological Sciences, University of North Carolina at Charlotte, Woodward Hall 380C, 9201 University City Blvd, Charlotte, NC 28223 USA; 4 Climate and Human Health Research Unit, Centre for Global Health Research, Kenya Medical Research Institute, Kisumu, Kenya; 5 Program in Public Health, College of Health Sciences, University of California, Irvine, CA 92697 USA; 6 Department of Medical Microbiology, College of Health Sciences, University of Ghana, Accra, Ghana

**Keywords:** Malaria risk factors, Case–control study, House design, Clinical malaria, Western Kenya

## Abstract

**Background:**

Understanding the complex heterogeneity of risk factors that can contribute to an increased risk of malaria at the individual and household level will enable more effective use of control measures. The objective of this study was to understand individual and household factors that influence clinical malaria infection among individuals in the highlands of Western Kenya.

**Methods:**

This was a matched case–control study undertaken in the Western Kenya highlands. Clinical malaria cases were recruited from health facilities and matched to asymptomatic individuals from the community who served as controls. Each participant was screened for malaria using microscopy. Follow-up surveys were conducted with individual households to collect socio-economic data. The houses were also checked using pyrethrum spray catches to collect mosquitoes.

**Results:**

A total of 302 malaria cases were matched to 604 controls during the surveillance period. Mosquito densities were similar in the houses of both groups. A greater percentage of people in the control group (64.6%) used insecticide-treated bed nets (ITNs) compared to the families of malaria cases (48.3%). Use of ITNs was associated with lower level of clinical malaria episodes (odds ratio 0.51; 95% CI 0.39–0.68; P < 0.0001). Low income was the most important factor associated with higher malaria infections (adj. OR 4.70). Use of malaria prophylaxis was the most important factor associated with less malaria infections (adj OR 0.36). Mother’s (not fathers) employment status (adj OR 0.48) and education level (adj OR 0.54) was important malaria risk factor. Houses with open eaves was an important malaria risk factor (adj OR 1.72).

**Conclusion:**

The identification of risk factors for clinical malaria infection provides information on the local malaria epidemiology and has the potential to lead to a more effective and targeted use of malaria control measures. These risk factors could be used to assess why some individuals acquire clinical malaria whilst others do not and to inform how intervention could be scaled at the local level.

## Background

Malaria remains a major public health problem in the tropical countries of the world, especially in sub-Saharan Africa [1]. Since the early 2000s, malaria interventions have been scaled up in sub-Saharan Africa with the goal to control and elimination of the disease. These interventions include the use of artemisinin-based combinations for treatment, and large-scale distribution of long-lasting impregnated net (LLINs) and indoor residual spraying (IRS). The interventions have largely been successful leading to a reduction of malaria incidence and prevalence in many parts of sub-Saharan Africa. However, in the last few years, there has been a resurgence of malaria in some areas [2–4]. In Western Kenya, malaria infections and vector densities have bounced back to their pre-intervention levels [2–5]. There is an urgent need to elucidate the risk factors that contribute to clinical malaria in different areas. Findings of this study would provide an explanation for the resurgence of malaria in sub-Saharan Africa.

Distance to mosquito breeding sites, household construction, the degree of household crowding and personal protection measures against mosquitoes are some of the well-known risk factors for malaria infections [6–9]. These factors can be influenced by differences in the environmental conditions, socio-economic status and cultural issues [10–12]. Most studies have shown an association between malaria disease prevalence and socio-economic status such as poor housing conditions, overcrowding, lack of knowledge about prevention of malaria, and low educational levels [9–12].

Information on the risk factors associated with the disease helps to understand the changing epidemiology over time and how different factors such as the application of interventions could be influenced [9, 13]. This is especially relevant to highland areas where malaria transmission is unstable and the risk of disease tends to be similar across all age groups, which have little to no immunity against *Plasmodium* sp. infections [14, 15]. Because malaria transmission could vary across climates, seasons, ecological zones, neighbouring villages, and even between neighbouring households [16], different areas may respond differently to interventions based on the associated risk factors. The present study investigated risk factors of clinical malaria at the individual and household levels in the highlands of Western Kenya.

## Methods

### Study site

**Fig. 1:**
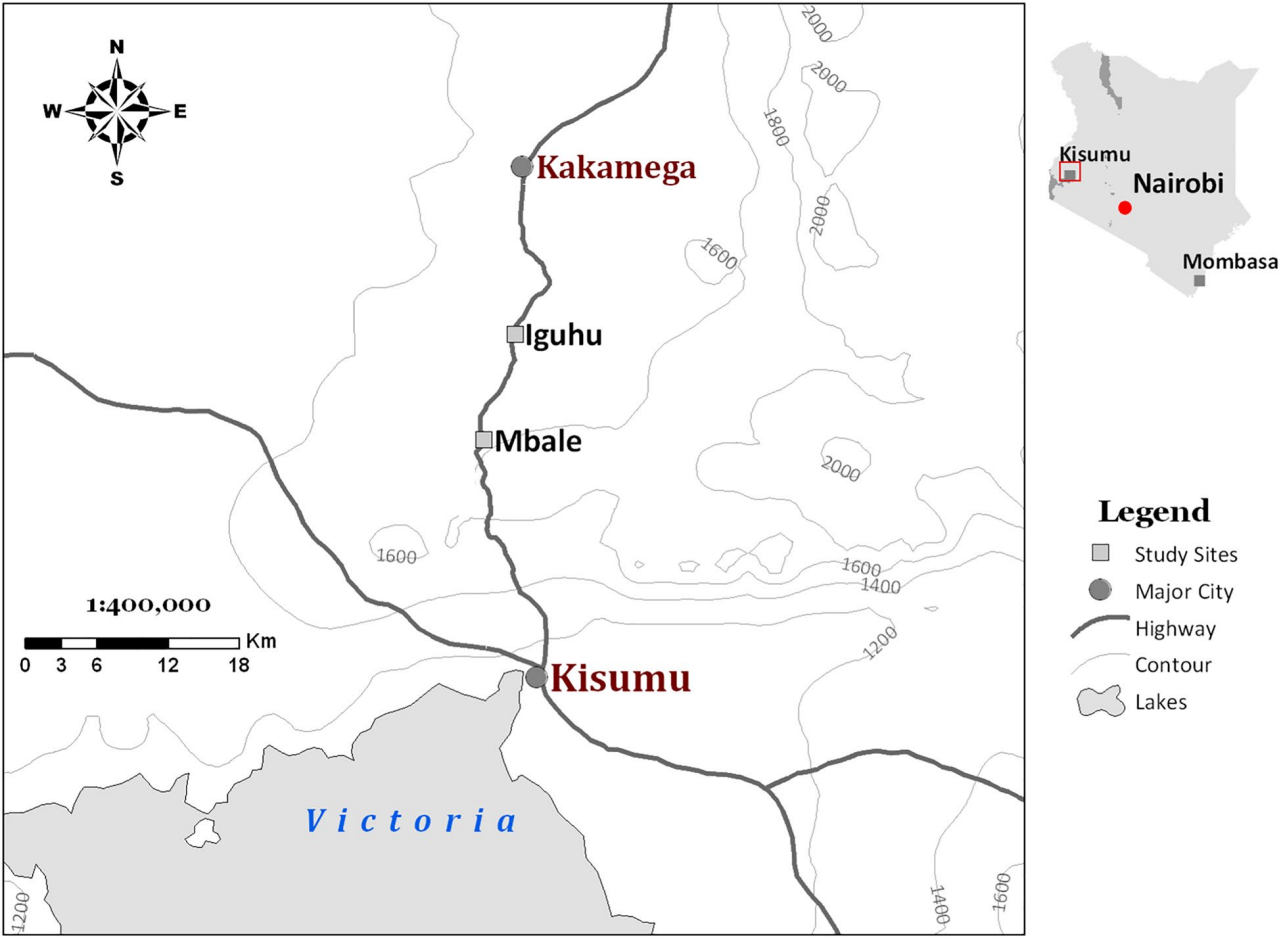
A map of the study sites

The study was conducted in Iguhu and Mbale, two highland areas in Western Kenya (Fig. 1). Iguhu (34°45′E, 0°10′N, 1430–1580 m above sea level) is located in Kakamega County and Mbale (34°74′E, 0°07′N, 1530–1690 m asl) in Vihiga County. Study participants were recruited from several clusters of villages in these two sites. Rainfall (annual average of 1977 mm) is seasonally bimodal, with the long rains occurring from March to June and the short rains from October to November. Average annual minimum and maximum temperatures are 13.8 °C and 28 °C, respectively. Malaria transmission is seasonal with peaks occurring 2–3 months after the peak rainy season in April–May, although the extent of the malaria burden varies considerably from year to year [17]. Vector breeding sites are known to be mostly found at the valley [18, 19]. *Plasmodium falciparum* is the predominant malaria parasite species in the study sites and it is transmitted by *Anopheles gambiae*, *Anopheles arabiensis* and *Anopheles funestus* [2, 18, 20, 21].

### Study participants, study design and data collection

This was a matched case–control study with 302 clinical malaria cases and 604 matched controls to explore association between participants/household risk factors and malaria infection undertaken from March 2012 to July 2013. There were 192 and 110 cases from Mbale and Iguhu, respectively. Confirmed malaria cases were recruited from patients who reported to the Iguhu and Mbale District Hospitals during the routine diagnosis of the out-patients. A malaria case was defined as patient confirmed with fever and malaria parasites identified in thick or thin blood smears who presented to the two local health centres with other malaria symptoms (chills, headache or vomiting). A finger prick blood sample was taken from each study participant from which a drop was placed on a microscope slide. Both thin and thick blood smears were prepared from the drop of blood on the slide. These were taken to the laboratory of the Kenya Medical Research Institute (KEMRI), in Kisumu and stained using Giemsa for microscopic examination. All participated patients were cross-checked microscopically for malaria parasite infection by the laboratory technician at KEMRI. Parasite density was scored against 200 leukocytes when the slide was positive; otherwise, the whole slide was carefully scanned before being declared negative. Parasite densities were converted to number of parasites per microliter of blood, assuming a leukocyte count of 8000 cells/μL [17]. Participants were recruited to the study regardless of age, gender, and socio-economic status. Enrollment of participants in the study was voluntary following a written consent/assent form. Infants below 5 months and adults above 45 years were excluded from the study due to the difficulty in obtaining a case and age-matched controls within the same locality.

Controls were recruited from the community and were matched to a case by age and neighborhood. Controls were individuals living in the same village as the cases and of almost the same age. All controls had no fever and other symptoms of malaria during the past 48 h prior to survey. These control individuals were also screened for parasite infection by microscope as described for case individuals.

### Household survey

Recruited cases and controls were followed up to their homes and a questionnaire was issued to them or their guardians in the case of children to collect socio-economic data. The altitude, longitude, and latitude of the households of all study participants were determined using a hand-held Trimble GeoExplorer Global Positioning System (GPS). Data on the type of household construction including the presence of eaves, screens, roof type, floor type, wall type and number of rooms in the house were recorded. A pre-tested questionnaire was administered to the household head (or spouse) of both cases and controls to obtain socio-economic and behavioral data such as number of occupants, level of education and occupation of both household head and spouse, travel history, health-seeking behaviour and household assets.

### Collection and identification of adult mosquitoes

Pyrethrum spray catches were done in the homes of both cases and controls to quantify the adult malaria vector densities in the houses. Mosquitoes were identified morphologically as *An. gambiae* sensu lato (s.l.), *An. funestus*, other *Anopheles,* and non-*Anopheles* [22], and characterized by gonotrophic stata (empty, blood-fed, gravid and half gravid female mosquitoes).

### Informed consent and ethical clearance

Ethical approval was obtained from the Institutional Review Boards (IRB) of the Kenya Medical Research Institute and the University of California at Irvine, USA. Written consent for adults and assent for minors were obtained from all participants after explaining the objectives and the methodology of the study to them.

### Data analysis

Data analysis was restricted to a comparison of cases and controls. Univariate analysis of the risk factors was conducted using logistic regression to estimate odds ratios (OR), adjusted to account for within-household clustering of cases. In multivariate analysis, conditional multiple-logistic regression was employed, here matching variables including matched group (one case and its two matched controls) and case versus control group (a covariate with case and control as values) were used as effect modifier. Student t-test was used to determine the differences in vector densities between case and control groups. Data analysis was conducted using SPSS.

## Results

### Socio-demographic factors

In this study, 48% of the surveyed malaria cases were males while 52% were females. The proportion of the controls matched to these cases was 46.7% and 53.3% males and females, respectively. Majority of the cases (60.9%) included in this study were aged below 5 years old. They were matched to 62.6% of the controls aged below 5 years old (Table 1).

**Table 1:** Socio-economic factors associated with the risk of malaria (univariate analysis) (n = 906)

Variable	Cases (%)	Controls (%)	Odds ratio	95% CI	P-value
Gender					
Male	48.0	46.7	Ref	0.72–1.25	0.708
Female	52.0	53.3	0.95		
Age (years)					
< 5	60.9	62.6	Ref		
≥ 5	39.1	37.4	1.07	0.81–1.42	0.632
Occupation status					
Father					
Jobless	6.3	7.9	Ref		
Farmer	47.0	49.7	1.20	0.68–2.11	0.538
Employed	46.7	42.4	1.39	0.79–2.46	0.254
Level of education					
Father					
None	2.3	2.0	Ref		
Primary	60.3	44.7	1.16	0.45–2.99	0.764
Secondary	32.5	43.9	0.63	0.24–1.66	0.348
Tertiary	5.0	9.4	0.45	0.15–1.34	0.127
Population in house					
< 5	38.7	38.6	Ref		
≥ 5	61.3	61.4	0.99	0.75–1.32	1.00

In terms of education and occupation status, families where the mother in the house was employed (logistic analysis, OR = 0.43; P = 0.04) and educated up to secondary school level (OR = 0.27; P < 0.0001) had lower chances of contracting malaria. However, the occupation status of father of the house (P = 0.54) and their corresponding level of education (P = 0.76) were not significant risk factors of malaria (Table 1). Both the cases (61.3%) and controls (61.4%) have similar household size with more than five family members living in the same house. The household size was not identified as a potential risk factor for malaria (OR = 0.99; P > 0.05; Table 1).

### Household characteristics

Most families of malaria cases and controls in the study sites lived in iron-roofed houses (89.7% and 94.7%, respectively) while the rest lived in grass-thatched houses. Compared to the grass-thatched roof, the iron roof was associated with a decrease in the risk of malaria (OR = 0.46; 95% CI 0.29–0.82; P < 0.005; Table 2). The majority of malaria cases (93.0%) and controls (84.9%) lived in houses constructed with mud walls, while the rest living in permanent houses made of cement blocks or bricks. Living in a permanent house made of cement blocks or bricks was significantly associated with lower malaria infections (OR = 0.42; 95% CI 0.26–0.69; P < 0.001; Table 2). In addition, most families of malaria cases and controls in the study site lived in houses with earthen floors (95% and 86.9%, respectively) while the rest had cemented floors. Families who lived in houses with cemented floor were well associated with a decrease in the risk of malaria (OR = 0.35; 95% CI 0.20–0.61; P < 0.001; Table 2). Living in a house with closed eaves was significantly associated with lower risk of getting malaria compared to living in houses with open eaves (OR = 0.46; P < 0.0001; Table 2). The size of houses and number of rooms per house were not malaria risk factors (OR = 1.07; 95% CI 0.68–1.70 and P = 0.76; Table 2).

**Table 2:** House-hold factors associated with the risk of malaria (univariate analysis) (n = 906)

Variable	Cases (%)	Controls (%)	Odds ratio (95% CI)		P-value
Roof					
Grass-thatch	10.30	5.30	Ref		
Iron-roof	89.70	94.70	0.49	0.29–0.82	0.0056
Wall					
Mud-wall	93.00	84.90	Ref		
Permanent	7.00	15.10	0.42	0.26–0.69	0.0005
Floor					
Earth floor	95.00	86.90	Ref		
Cemented	5.00	13.10	0.35	0.20–0.61	0.0002
Eaves					
Open eaves	49.00	32.10	Ref		
Closed eaves	51.00	67.90	0.49	0.37–0.65	< 0.0001
Screens					
Yes	1.00	0.80	Ref		
No	99.00	99.20	0.83	0.20–3.50	1.00
No. of rooms					
> 5	9.90	10.60	Ref		
≤ 5	90.10	89.40	1.07	0.68–1.70	0.7642

### Vector density

**Fig. 2:**
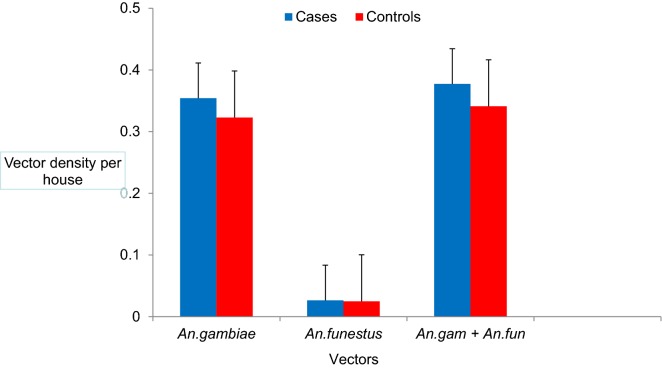
Mean density of vectors in the households of cases and controls

There was no significant difference in vector density in the houses of malaria case groups (0.38 ± 0.058 vectors/house) compared to the control group (0.34 ± 0.078 vectors/house; t = 1.96; df = 895; P = 0.71; Fig. 2). In both study groups, a higher density of *An. gambiae* s.l. (0.36 ± 0.057 vectors/house) than *An. funestus* (0.02 ± 0.009 vectors/house) was observed.

### Malaria and mosquito prevention methods

Insecticide-treated bed nets usage was higher in control group (64.6%) as compared to case group (48.3%), understandably, malaria risk was lower in families using ITNs compare to those who did not use ITNs (OR = 0.51; 95% CI 0.39–0.68; P < 0.0001). The proportion of families that use alternative mosquito prevention measures such as aerosol sprays, herbs, and mosquito coils was higher among the control groups (63.1%) compared to those of the cases group (44.0%). Those who used other mosquito prevention measures had lower malaria risk compared to those who did not use the prevention measures (OR = 0.46; 95% CI 0.35–0.61; P < 0.0001).

There were more individuals in the control group (45.7%) used prophylactic anti-malarials compared to the cases group (22.2%). The use of prophylaxis was significantly associated with lower malaria risks (OR = 0.34; 95% CI 0.25–0.46 P < 0.0001; Table 3). Most families of both malaria cases (98.7%) and controls (92.7%) sought diagnosis and treatment from government health facilities. However, the percentage of families that resorted to private health facilities was lower among malaria cases (1.3%) compared to control groups (7.3%; Table 3). The health facility from which therapy was sought was associated with lower risk of malaria infections (OR = 0.17; 95% CI 0.06–0.48; P < 0.001; Table 3).

**Table 3:** Malaria and mosquito prevention factors associated with the risk of malaria (univariate logistic regression)

Variable	Cases	Controls	OR	95% CI	P-value
ITN use					
No	51.7	35.4	Ref		
Yes	48.3	64.6	0.51	0.39–0.68	< 0.0001
Malaria prophylaxis					
No	77.8	54.3	Ref		
Yes	22.2	45.7	0.34	0.25–0.46	< 0.0001
Mosquito prevention					
No	56.0	36.9	Ref		
Yes	44.0	63.1	0.46	0.35–0.61	< 0.0001
Healthcare services					
Govt facility	98.7	92.7	Ref		
Private facility	1.3	7.3	0.17	0.06–0.48	0.0002

### Multiple regression analysis

The risk of malaria was significantly reduced for families that use malaria prophylaxis and those where the mother of the houses occupation was farming. The risk of malaria was high in families where the mother was not educated or whose income was less than 2000 Kenya shillings (USD 25 at the time of data collection) per month (Table 4).

**Table 4:** Multivariate conditional logistic regression analysis of individual and household factors associated with the risk of malaria in Western Kenya

Term	Estimate (95% CI)	Chi-square	Prob > ChiSq	Odds ratio^a^
Occupation father (farmer)	0.43 (0.24, 0.61)	19.78	< 0.0001	2.32
Mother edu. (none)	0.31 (0.06, 0.56)	5.82	0.0159	1.85
Income **< **2000	0.83 (0.59, 0.96)	65.52	< 0.0001	4.70
Open eaves	0.27 (0.10, 0.44)	9.39	0.0022	1.72
Occupation mother (not farmer)	− 0.36 (− 0.61, − 0.12)	8.65	0.0033	0.48
Malaria prophylaxis	− 0.52 (− 0.70, − 0.34)	30.76	< 0.0001	0.36

^a^OR > 1 indicating higher malaria risk and OR < 1 indicating lower malaria risk

## Discussion

Malaria epidemiology is affected by biotic, abiotic and socio-economic factors. The distribution of the disease in Western Kenya is heterogeneous and can vary greatly between villages and households [12]. This study showed that, to a great extent, abiotic and socio-economic factors such as housing design and structure as well as the use of mosquito preventing methods and prophylaxis affect malaria infection and the incidence of clinical malaria.

The type of houses had an impact on the incidence of clinical malaria. In the present study, majority of the people with clinical malaria lived in houses that were made of mud, had grass-thatched houses or houses with opened eaves. These types of house design or construction likely allow mosquitoes to fly in and bite [23], and thus offer less protection compared to those made of cement blocks and iron-roofing sheets with closed eves. The house characteristics have been shown to be a risk factor for incidence of malaria [24].

Several studies have shown that the lack of mosquito prevention methods such as bed nets, screen windows, and doors, burning of coils and aerosol sprays is a risk factor for clinical malaria incidence [7, 12]. These study findings support such notion and indicated that the control group had fewer malaria infections because a larger proportion of individuals used one or more of these mosquito prevention measures to avoid contacts with mosquitoes than in the cases group. The socio-economic status of a family relates directly to the affordability of mosquito prevention methods at the household level. With the exception of insecticide-treated bed nets that are given for free, all other mosquito prevention methods have to be purchased. Therefore, whether the household head or spouse is employed or self-employed would determine the type of mosquito prevention method(s) used by each family. Furthermore, the educational level of the household head or spouse would reflect if they understand the risk of malaria and take necessary knowledge-based precaution to avoid mosquito bites.

The study findings underscore the complexity of highland malaria transmission. Previous studies have assessed some of the malaria risk factors that we evaluated, but this study adds to these earlier reports by investigating participants/household malaria risk factors and vector densities. Given that in this study individuals were not followed overtime, this may have an effect on vector densities as the number of mosquitoes indoors depends on several factors such as seasonality and use of interventions. The identification of risk factors for clinical malaria infection provides information on the local malaria epidemiology and has the potential to lead to a more effective strategy of malaria control. Although the impact of host-parasite interactions and insecticide resistance in the study area were not taken into account, the study findings indicate that a number of socio-economic and abiotic variables alone or in combination could affect the risk of acquiring *Plasmodium* infection in highland areas with malaria transmission is heterogenous. These factors explain why some individuals acquire clinical malaria whilst others do not.

## Conclusion

This study confirmed that educational status, occupation and house structure were important risk factors for malaria infection, and these findings were consistent with previous reports in East Africa. Further studies with strong entomology components should be conducted to generate more evidence, particularly in low transmission settings similar to the current highland region.

## Data Availability

Data for the manuscript are available in the Kenya Medical Research data repository to anyone who might need it.

## Abbreviations

ITN: insecticide-treated bed nets
LLIN: long-lasting insecticide-impregnated nets
IRS: indoor residual spray
ACT: artemisinin-based combination therapy
IRB: Institutional Review Board
CI: confidence interval
OR: odds ratio
GPS: Global Positioning System
